# Investigation of Bacteremia due to *Aeromonas* Species and Comparison with That due to Enterobacteria in Patients with Liver Cirrhosis

**DOI:** 10.1155/2011/930826

**Published:** 2011-12-29

**Authors:** Toru Shizuma, Chiharu Tanaka, Hidezo Mori, Naoto Fukuyama

**Affiliations:** Department of Physiology, School of Medicine, Tokai University, 143 Shimokasuya, Isehara, Kanagawa 259-1193, Japan

## Abstract

*Background*. The role of *Aeromonas* species (sp.) in bacteremia in Japanese patients with liver cirrhosis is poorly understood. *Aim*. To establish the importance of *Aeromonas* sp. as a cause of bacteremia in patients with liver cirrhosis. *Methods*. Clinical and serological features and short-term prognosis were retrospectively investigated and compared in Japanese patients with bacteremia due to *Aeromonas* sp. (*n* = 11) and due to enterobacteria (*E. coli, Klebsiella* sp., and *Enterobacter* sp.) (*n* = 84). *Results*. There were no significant differences in patients' clinical background, renal dysfunction, or short-term mortality rate between the two groups. However, in the *Aeromonas* group, the model for end-stage liver disease (MELD) score and Child-Pugh score were significantly higher than in the enterobacteria group. *Conclusion*. These results indicate that the severity of liver dysfunction in *Aeromonas*-induced bacteremia is greater than that in enterobacteria-induced bacteremia in Japanese patients with liver cirrhosis.

## 1. Introduction

The widespread use of cephem antibiotics and the increase in the number of patients with indwelling catheters have led to an increase in the proportion of Gram-positive bacteria among pathogenic bacteria causing bacteremia in hospitalized patients in Japan since the early 1980s [1]. Nevertheless, Gram-negative bacteria, including enterobacteria such as *E. coli* and *Klebsiella* sp., are still the most important causes of bacteremia in liver cirrhosis patients [1–5]. However, the source of infection is often unknown in bacteremia of liver cirrhotic patients, and the pathogenic bacteria can rarely be identified from clinical findings alone. There has been no previous report of a comparative study on the roles of *Aeromonas* sp. and other bacteria in bacteremia of liver cirrhosis patients. Therefore, we conducted a retrospective study of liver cirrhotic patients in Japan to examine the importance of *Aeromonas* as a cause of bacteremia, compared with enterobacteria (*E. coli*, *Klebsiella* sp., and *Enterobacter* sp.).

## 2. Materials and Methods

### 2.1. Subjects and Methods

The subjects of this study were patients hospitalized for liver cirrhosis in the Department of Gastroenterology, Tokyo Women's Medical College, Tokyo (1991–2003), Nagashio Hospital, Tokyo (1996–2004), Kikuna Memorial Hospital, Kanagawa (2007–2009), or the Department of Gastroenterology, International University of Health and Welfare Hospital, Tochigi (2007–2010), all of which are located in the Kanto district of Japan.

Patients with bacteremia were included in this study, regardless of whether or not septicemia was present. Patients whose blood cultures showed multiple types of bacteria were excluded from the study. For blood culture, one or several samples of venous or arterial blood had been aseptically collected and inoculated into aerobic (BACTEC Plus Aerobic/F Culture Vial) and anaerobic (BACTEC Plus Anaerobic/F Culture Vial) culture bottles. Culture was conducted with shaking for seven days using a dedicated blood culture system (Becton, Dickinson Co., USA).

Patients with diagnoses of spontaneous bacterial peritonitis (SBP), infectious pleural effusion, peritonitis carcinomatosa, and hemorrhagic ascites, such as hepatocellular carcinoma rupture, were also excluded from the study. SBP was diagnosed regardless of the presence of bacteria in the ascitic fluid in cases where the neutrophil count in ascitic fluid was 500/mm^3^or more. In cases having subjective and objective symptoms of SBP, a neutrophil count between 250/mm^3^ and 500/mm^3^ was considered sufficient [6].

Patients whose blood cultures were positive for any of *E. coli*, *Klebsiella* sp., or* Enterobacter* sp. were assigned to the enterobacterial bacteremia group, and those blood cultures were positive for *Aeromonas* sp. were assigned to the *Aeromonas* bacteremia group. Patients' backgrounds, clinical and serological findings, and short-term (within one month) prognoses were compared between the two groups. Hematological test data including the MELD score and Child-Pugh score obtained at the time of blood culture are expressed as mean ± standard deviation (SD).

### 2.2. Statistical Analysis

Statistical analysis as conducted using an unpaired *t*-test to compare the ages of patients and hematological test data in the two groups, and a contingency table was used to compare ratios in patients' background factors and mortality rates. Differences were considered significant at the level of *P* < 0.05 in all statistical analysis.

## 3. Results

### 3.1. Rates of Pathogenic Bacteria in Bacteremia

Among the liver cirrhosis patients with bacteremia in this study, 21.3% (44/207) showed blood cultures positive for *E. coli*, 17.9% (37/207) for *Klebsiella* sp., 2.4% (5/207) for *Enterobacter* sp., and 5.3% (11/207) for *Aeromonas* sp. (Table 1). Excluding two cases in which multiple bacteria were detected, we obtained 84 cases involving enterobacteria (43 *E. coli*, 36 *Klebsiella* sp., and 5 *Enterobacter* sp.), and 11 cases involving *Aeromonas* sp.

### 3.2. Species and Onset Period of Bacteremia due to Aeromonas

There were 9 cases in which *A. hydrophila *was detected and 2 cases in which *A. veronii* biovar sobria (identified as *A. sobria* at the time) was detected. Of these 11 cases, 4 cases had a summer onset (June through August), while 3 had a winter onset (December through February). None of the patients involved was known to have eaten raw fish.

### 3.3. Comparison of Background Factors and Clinical Findings in the Two Groups

No significant difference was observed in the backgrounds of the two groups, including age, presence or absence of hepatocellular carcinoma or diabetes mellitus, occurrence of ascites or acute diarrhea, frequency of shocks, rate of complication with SBP, or history of gastrointestinal hemorrhage within two weeks of onset (Table 2). Moreover, no patients with prior episodes of cholecystitis were present in the *Aeromonas* bacteremia group in this study, although 2 out of 11 had gallstones.

### 3.4. Comparison of Hematological Test Data in the Two Groups

The Child-Pugh score was significantly (*P* < 0.01) higher in the *Aeromonas *bacteremia group (13.3 ± 0.81) versus the enterobacterial bacteremia group (11.2 ± 1.93). The MELD score [7] was also significantly (*P* < 0.01) higher in the *Aeromonas *bacteremia group (26.1 ± 1.86) versus the enterobacterial bacteremia group (21.9 ± 2.22). The two groups showed no significant difference in albumin levels, ammonia levels, BUN levels, or creatinine levels (Table 3).

### 3.5. Comparison of Short-Term Prognosis

There was no significant difference between the two groups in terms of the one-month mortality rate, which was 41.7% (35/84) in the enterobacterial bacteremia group and 54.5% (6/11) in the *Aeromonas* bacteremia group.

## 4. Discussion

Patients with liver cirrhosis are liable to suffer from bacterial infection because of impaired function of the hepatic reticuloendothelial system and neutrophils, an influx of bacteria into the systemic circulation due to portal-caval shunts or a decreased opsonic effect in the ascites and the like [1, 3, 8]. The onset of bacterial infection in patients hospitalized with liver cirrhosis is reported to be 30% to 60% [6, 9], and bacteremia has been reported as occurring in 3.5% to 8.8% [2, 3, 10].

The infection focus is often unknown in bacteremia of liver cirrhosis patients [1]. However, breakdown of the intestinal mucosa and changes in the intestinal flora, especially enterobacteria, as well as bacterial translocation to the mesenteric lymph nodes from the bowel, are thought to be involved in the onset of bacteremia and SBP [8]. Bacterial translocation has been reported to be common in patients with Child-Pugh class C cirrhosis [11], and it is easily induced in animal models of severe liver disease [12]. In addition, in patients with liver cirrhosis, more severe hepatic dysfunction is generally considered to be associated with a higher rate of onset of bacteremia [1, 2], and patients who develop bacteremia have a worse prognosis than cases not complicated by bacteremia [1, 2, 5]. However, although there have been reports comparing cases of SBP involving *Aeromonas* sp. and other bacterial species [13], the present report is the first to describe a comparative study on *Aeromonas* bacteremia and bacteremia due to other bacterial species in liver cirrhosis patients.

*Aeromonas* sp., which are Gram-negative, are found throughout the natural world (in water and soil), and three types—*A. hydrophila*, *A. caviae*, and *A. veronii* biovar sobria (formerly known as *A. sobria*)—are pathogenic to humans [14, 15]. *Aeromonas* bacteremia is rare in healthy people, but is often found in cases of decompensated liver cirrhosis, hematological malignancy, and hepatobiliary infection [14, 16–18], and has been quite well studied in Taiwan and Republic of Korea among East Asian countries [2, 15–19]. The origin of the bacteremia in *Aeromonas* bacteremia with decompensated liver cirrhosis is often unknown. In our study, there were no patients in whom hepatobiliary infection appeared to be the focus of bacteremia in the *Aeromonas* bacteremia group, although 2 out of 11 had gallstones.

Sources of *Aeromonas* infection include raw fish or unboiled water [14, 19]. The proportion of *Aeromonas* among bacteremia cases in Japan is small, probably because of the general availability of uncontaminated drinking water in Japan, and because liver cirrhosis patients are advised not to eat raw fish. Although *Aeromonas* bacteremia, along with *Vibrio* sp. infection, tends to occur more frequently in the summer [13, 15], the 11 cases of *Aeromonas* bacteremia in this study reported no history of eating raw fish, or of skin or soft tissue infection, and the incidence was not greater in summer.

In this study, 90.9% (10/11) of the *Aeromonas* bacteremia patients had severe liver cirrhosis with a Child-Pugh score of 13 or above, which was significantly higher than the score in enterobacterial bacteremia. Also, although Choi et al. found no significant difference in SBP cases, they reported a higher number of Child-Pugh C cases with *Aeromonas* than with other bacterial species [13], and the average Child-Pugh score of *Aeromonas* bacteremia in the study by Ko et al. was also 12 points or higher [18].

Moreover, the MELD score was also significantly higher in the* Aeromonas* bacteremia group than in the enterobacterial bacteremia group in our study. Therefore, *Aeromonas* bacteremia may be more likely to arise in liver cirrhosis patients with severe hepatic dysfunction.

Bacterial infection is involved in a quarter of mortalities among cirrhosis patients [9, 20], and the mortality rate among patients with complications arising from bacterial infection is markedly higher [1, 21, 22]. Previous reports have described short-term mortality rates of 27% to 60% in liver cirrhosis complicated by bacteremia [1, 3, 4, 21], though slightly different definitions of short-term mortality have been employed. Recently, the mortality rate was reported to be 42.2% among 1437 cirrhosis patients [21], and in our study the overall mortality rate was 43.2% (41/95) in the two groups combined.

It has been suggested that septic shock [13, 18] or the onset of renal impairments, such as hepatorenal syndrome [1, 23], plays an important role in determining the prognosis of *Aeromonas* infection in liver cirrhosis. However, we found no significant difference in the short-term mortality rate, renal dysfunction, and percentage of shock between the enterobacterial group and *Aeromonas* bacteremia group in this study. Nevertheless, our results indicate that the severity of liver dysfunction in *Aeromonas*-induced bacteremia is greater than that in enterobacteria-induced bacteremia in patients with liver cirrhosis.

## Figures and Tables

**Table 1:** Microorganisms isolated from blood cultures in patients with liver cirrhosis.

Microorganisms	Number	%
Gram-positive bacteria	76	36.7%
MRSA	29	14.0%
MSSA	21	10.1%
*Staphylococcus epidermidis *	10	4.8%
*Enterococcus* sp.	7	3.4%
*Streptococcus* sp.	5	2.4%
Others	4	1.9%
Gram-negative bacteria	123	59.4%
*Escherichia coli *	44	21.3%
*Klebsiella* sp.	37	17.9%
*Pseudomonas* sp.	19	9.2%
*Aeromonas* sp.	11	5.3%
*Enterobacter* sp.	5	2.4%
Others	7	3.4%
Others	8	3.9%

Total	207	100%

MRSA: methicillin resistant *Staphylococcus aureus.*

MSSA: methicillin sensitive *Staphylococcus aureus.*

**Table 2:** Comparison of background factors and clinical findings in the two groups.

	Aeromonas bacteremia	Enterobacterial bacteremia	*P* value
Ages	61.2 ± 4.5	62.3 ± 10.8	*P* > 0.05
Hepatocellular carcinoma	36.4% (4/11)	47.6% (40/84)	*P* > 0.05
Diabetes	27.3% (3/11)	28.6% (24/84)	*P* > 0.05
Ascites	100% (11/11)	90.5% (76/84)	*P* > 0.05
Diarrhea	9.1% (1/11)	4.8% (4/84)	*P* > 0.05
Shock (BP < 80 mmHg)	36.4% (4/11)	15.5% (13/84)	*P* > 0.05
SBP	9.1% (1/11)	6.0% (5/84)	*P* > 0.05
Gastrointestinal bleeding within 2 weeks	9.1% (1/11)	19.0% (16/84)	*P* > 0.05

SBP: spontaneous bacterial peritonitis.

**Table 3:** Comparison of laboratory findings in the two groups.

	*Aeromonas* bacteremia	Enterobacterial bacteremia	*P* value
PT (%)	36.3 ± 15.9	42.7 ± 23.2	*P* > 0.05
Albumin (g/dL)	2.40 ± 0.42	2.47 ± 0.49	*P* > 0.05
T-bil. (mg/dL)	9.7 ± 6.4	7.8 ± 8.9	*P* > 0.05
NH3 (*μ*g/dL)	97 ± 37	89 ± 39	*P* > 0.05
BUN (mg/dL)	27.1 ± 8.9	27.5 ± 9.7	*P* > 0.05
Creatinine (mg/dL)	1.32 ± 0.55	1.36 ± 0.59	*P* > 0.05
Child-Pugh score	13.3 ± 0.81	11.2 ± 1.93	*P* < 0.01
MELD score	26.1 ± 1.86	21.9 ± 2.22	*P* < 0.01

T-bil.: total bilirubin.

MELD: model for end-stage liver disease.
